# The prostate-specific membrane antigen holds potential as a vascular target for endogenous radiotherapy with [^177^Lu]Lu-PSMA-I&T for triple-negative breast cancer

**DOI:** 10.1186/s13058-024-01787-9

**Published:** 2024-02-20

**Authors:** Amelie Heesch, Alexandru Florea, Jochen Maurer, Pardes Habib, Laura S. Werth, Thomas Hansen, Elmar Stickeler, Sabri E. M. Sahnoun, Felix M. Mottaghy, Agnieszka Morgenroth

**Affiliations:** 1 Department of Nuclear Medicine, University Hospital RWTH Aachen, Pauwelsstraße 30, 52074 Aachen, Germany; 2 Department of Radiology and Nuclear Medicine, Maastricht University Medical Center (MUMC+), 6202 Maastricht, The Netherlands; 3 School for Cardiovascular Diseases (CARIM), Maastricht University Medical Center (MUMC+), 6202 Maastricht, The Netherlands; 4 Department of Obstetrics and Gynecology, University Hospital RWTH Aachen, 52074 Aachen, Germany; 5 Center for Integrated Oncology (CIO), Aachen, Bonn, Cologne, Düsseldorf (ABCD), Germany; 6 Department of Neurosurgery, School of Medicine, Stanford University, Stanford, USA

**Keywords:** Triple-negative breast cancer, Prostate-specific membrane antigen, Endogenous radiotherapy, Anti-angiogenic therapy, Orthotopic xenograft

## Abstract

**Introduction:**

Overexpression of prostate-specific membrane antigen (PSMA) on the vasculature of triple-negative breast cancer (TNBC) presents a promising avenue for targeted endogenous radiotherapy with [^177^Lu]Lu-PSMA-I&T. This study aimed to assess and compare the therapeutic efficacy of a single dose with a fractionated dose of [^177^Lu]Lu-PSMA-I&T in an orthotopic model of TNBC.

**Methods:**

Rj:NMRI-*Foxn1*^*nu/nu*^ mice were used as recipients of MDA-MB-231 xenografts. The single dose group was treated with 1 × 60 ± 5 MBq dose of [^177^Lu]Lu-PSMA-I&T, while the fractionated dose group received 4 × a 15 ± 2 MBq dose of [^177^Lu]Lu-PSMA-I&T at 7 day intervals. The control group received 0.9% NaCl. Tumor progression was monitored using [^18^F]FDG-PET/CT. Ex vivo analysis encompassed immunostaining, TUNEL staining, H&E staining, microautoradiography, and autoradiography.

**Results:**

Tumor volumes were significantly smaller in the single dose (*p* < 0.001) and fractionated dose (*p* < 0.001) groups. Tumor growth inhibition rates were 38% (single dose) and 30% (fractionated dose). Median survival was notably prolonged in the treated groups compared to the control groups (31d, 28d and 19d for single dose, fractionated dose and control, respectively). [^177^Lu]Lu-PSMA-I&T decreased the size of viable tumor areas. We further demonstrated, that [^177^Lu]Lu-PSMA-I&T binds specifically to the tumor-associated vasculature.

**Conclusion:**

This study highlights the potential of [^177^Lu]Lu-PSMA-I&T for endogenous radiotherapy of TNBC.

**Graphical abstract:**

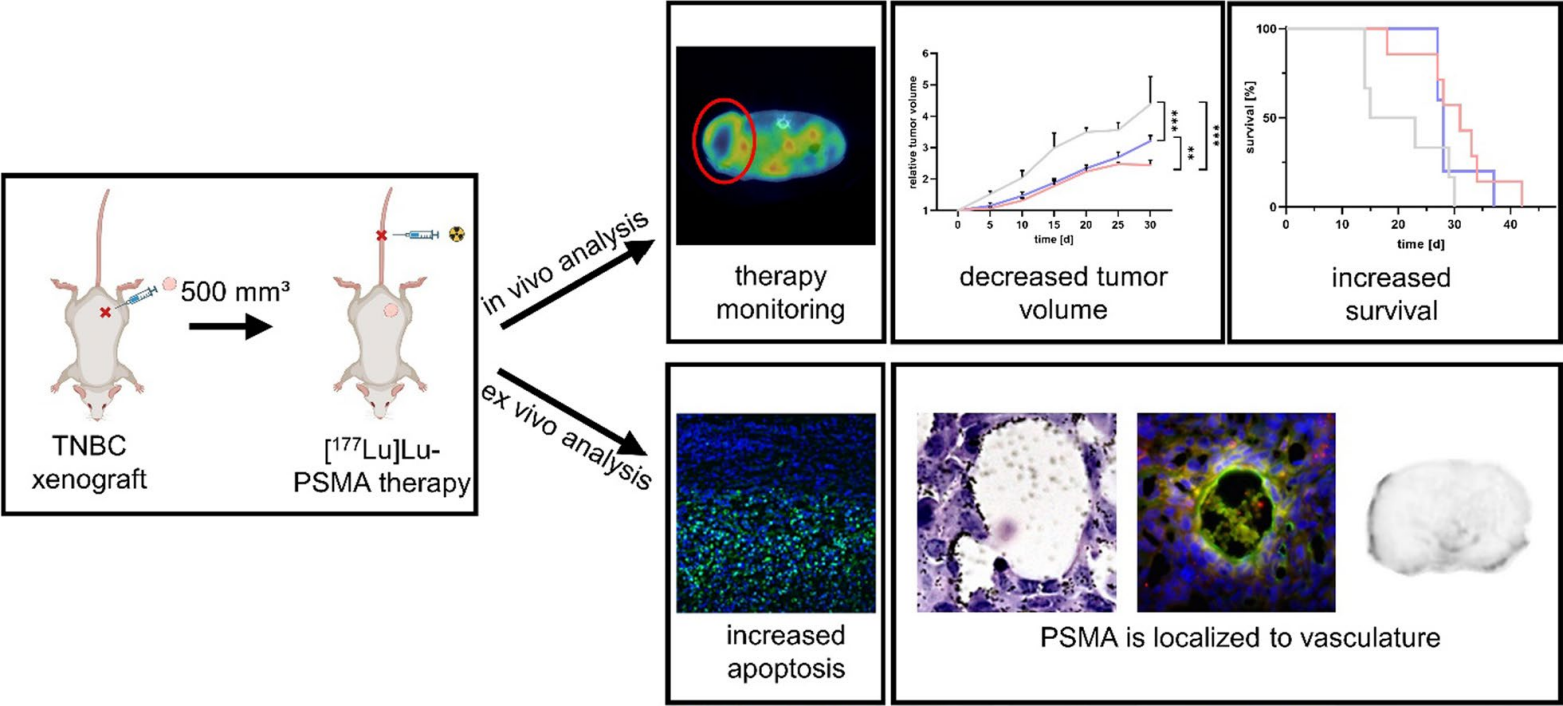

**Supplementary Information:**

The online version contains supplementary material available at 10.1186/s13058-024-01787-9.

## Introduction

Triple-negative breast cancer (TNBC) is an exceptionally aggressive breast cancer and accounts to approximately 10–20% of all breast cancers, it remains a formidable clinical challenge due to the dearth of therapeutic interventions [1]. Currently, cytotoxic chemotherapy remains the prevailing clinical standard. Although the initial response is good, more than 50% of patients experience a relapse after 3–5 years and the mortality rates are higher compared to other types of breast cancer [2]. One reason contributing to the poor prognosis is the population of ALDH1-expressing stem cells, which is closely associated with therapy resistance [3]. Around 46% of the patients will develop distant metastasis highlighting the aggressiveness of this subtype [4]. The high metastatic potential is closely associated with the especially high microvascular density found in TNBC [5].

The underlying process of this microvasculature formation is angiogenesis, which plays a major role in tumor growth and metastasis and is a promising research subject. Generally, the inhibition of tumor growth via anti-antiangiogenic pharmaceuticals presents one potential treatment strategy. For this, vascular endothelial growth factor (VEGF) receptor antagonists are used to block the receptor’s catalytic function. However, several phase III trials like AVADO [6], RIBBON-1 [7] and RIBBON-2 [8] using bevacizumab in combination with different chemotherapeutica demonstrated no significant change in overall survival in metastatic breast cancer [9]. Just recently prostate-specific membrane antigen (PSMA) came into focus as a potential therapy target as it is overexpressed on the vasculature of many solid tumors including TNBC [10]. Several in vitro studies demonstrated PSMA expression on human umbilical vein endothelial cells (HUVECs) after co-culture with breast cancer cells or tumor-conditioned media [11–13].

Clinically, radiolabeled PSMA-ligands are used for treatment of castration-resistant prostate cancer since PSMA is highly overexpressed on the tumor cells [14]. [^177^Lu]Lu-PSMA induces high response rates in this tumor entity with prolonged overall survival and decreased tumor size [15]. Although [^177^Lu]Lu-PSMA-I&T is excreted via the kidneys, the reported renal toxicity is rather low [16].

In recent in vitro studies we have demonstrated that PSMA is expressed on tumor-associated endothelial cells, and to some amount on the tumor cells in TNBC [13].

## Material and methods

### ***Radiosynthesis of [***^***177***^***Lu]Lu-PSMA-I&T***

[^177^Lu]Lu-PSMA-I&T was produced by a GMP approved clinical routine procedure primarily used for patient care. In summary, a cassette synthesizer type GRP 3 V (Scintomics, Germany) was used with cassettes (ABX, Germany) dedicated to the radionuclide (SC-05 for ^177^Lu using acetate buffer during labeling). Up to 2 GBq (< 1 mL) ^177^LuCl_3_ (ITM, Germany) was transferred to the reactor containing precursor PSMA-I&T and acetate buffer. After 20 min reaction at 100 °C, the solution was quenched by a saline solution containing DTPA. Radiochemical purities and yields were > 95% (measured with radio-HPLC).

### ***Procurement of [***^***18***^***F]FDG***

An [^18^F]FDG injection solution (GLUSCAN®, Advanced Accelerator Applications, Saint-Genis-Pouilly, France) was purchased with a volume activity of 600 MBq/mL at the time of calibration.

### Cell culture

The MDA-MB-231 cell line (ATCC, USA/VA) was cultured in DMEM (Pan Biotech, Germany) supplemented with 10% fetal bovine serum and 1% penicillin/streptomycin (Pan Biotech, Germany) at 37 °C and 5% CO_2_. Cells were tested biweekly for mycoplasma contamination.

### Tumor model, study design and animal care

Female Rj:NMRI-*Foxn1*^*nu/nu*^ mice (Janvier, France) at 6–8 weeks of age were used for orthotopic TNBC xenograft implantation. Animals were housed at 20–24 °C in a 12 h daylight cycle and given at least 7 days for acclimatization before the start of the experiment. For each mouse, 5 × 10^6^ MDA-MB-231 were resuspended in a 10 µL culture media/10 µL matrigel mixture (Corning, USA/NY). Mice were inoculated in the mammary fat pad on the right flank. Tumor growth was monitored daily using caliper measurements. Tumor volume was calculated daily according to the following formula assuming an ellipsoid shape:

$${\text{V}}_{{{\text{tumor}}}} = {\text{ length}}_{{{\text{tumor}}}} \times \, \left( {{\text{width}}_{{{\text{tumor}}}} } \right)^{{2}} \, \times \, 0.{52}$$

In addition to the daily caliper measurements, image analysis of [^18^F]FDG-PET/CT based tumor volumes was blinded to avoid bias. Animals were finalized upon reaching a humane endpoint for the therapy study (*i.e.*, 15 mm tumor diameter or 1500 mm^3^ tumor volume, exulceration, ≥ 20% weight loss). From 24 animals, which were used in this study, 6 mice were not considered for the quantification due to reaching an unusual early end point (tumor diameter 15 mm, but tumor volume < 1000 mm^3^).

Animals were randomly distributed into 3 groups: Control (n = 6), [^177^Lu]Lu-PSMA-I&T single dose (60 MBq) (n = 7) or fractionated dose (4 × 15 MBq) (n = 5). Randomization was done based on equivalent distribution of tumor volumes.

### Therapy study

Under 1.5–2.5% isoflurane in medical grade compressed air at 0.8 L/min, the lateral tail vein was injected with [^177^Lu]Lu-PSMA-I&T (single dose: 60 ± 5 MBq once, fractionated dose: 15 ± 2 MBq every 7 days for 4 weeks) diluted with 0.9% NaCl to a total volume of 50 µL. The administered dose was calculated by subtraction of decay-corrected syringe activity post-injection from pre-injection activity.

### Small animal PET/CT imaging

Initially the animals received an intraperitoneal injection with 10 MBq of [^18^F]FDG diluted with 0.9% NaCl to a total volume of 100 µL; 30 min later, each animal was placed on the scanner bed and the CT scan was initiated under 1.5–2.5% isoflurane in medical grade compressed air at 0.8 L/min. All mice were imaged with a small animal PET/SPECT/CT system (Tri-umph®II, Northridge Tri-Modality Imaging, Inc., Chatsworth, USA; x-Cube and γ-Cube, Molecubes, Gent, Belgium), however only the PET and CT modalities were used for this study. The exposure settings used were as follows: 130 uA, 75 kVp, 230 ms exposure time, and 360° rotation with 512 views; the duration of the CT scans was ~ 5 min. A dynamic 30 min PET scan was initiated at the end of the CT scan. The CT had an axial field of view of 91.1 mm and a PET of 112 mm. During the scans, the isoflurane concentration was adapted to achieve a respiratory rate between 75 and 50 breaths per minute.

The CT images were reconstructed using a Feldkamp filtered back projection reconstruction process to a voxel size of 0.154 × 0.154 × 0.154 mm in a 592 × 592 × 560 matrix. Using vendor software, the CT values were converted into Hounsfield units (HU) using the following formula [17]:

$${\text{HU }} = { 1}000 \, \times \, \left( {\mu_{{\text{t}}} - \mu_{{\text{w}}} } \right)/\mu_{{\text{w}}}$$

where *μ*_w_ is the linear attenuation coefficient of the water and *μ*_t_ is the linear attenuation coefficient of the tissue. The PET data were reconstructed using a 3D ordered-subset expectation maximization (i.e., OSEM-3D with three iterations and eight subsets) with a maximum a posteriori probability algorithm (30 iterations) into a 240 × 240 × 192 image matrix (resulting in final voxel dimensions of 0.25 × 0.25 × 0.597 mm). PET normalization, CT attenuation correction, and CT scatter correction were applied to all PET reconstructions. The PET images were automatically aligned to the CT using a custom-made transformation in PMOD software package from a capillary phantom. For the 2D image presentation, the PFUS tool from Pmod was used. To exclude the bed and other objects from the CT image, an automatic isocontour detection around the mouse using -500 HU as a minimal threshold on the coronal view was used. The newly created images were used to show exemplary CT by capturing with a window from − 1000 to + 1000 HU. PET images were captured using a window from 0 to 2.6 SUV_bw_. For the fusion, the images co-registered PET/CT scans were used.

### TUNEL staining

After finalization, excised organs (*i.e.*, tumor, kidney, liver, spleen, colon, small intestine) were fixed with 4% PFA overnight at 4 °C. For cryopreservation, the organs were kept for 2–3 days at 4 °C in 30% sucrose. Cryosections (6 μm) of tumor and organs were made using a cryostat (Leica, CM3050S). The TUNEL staining procedure was carried out with the in situ cell death detection kit accordingly to the manufacturer’s instructions (Roche, Switzerland). Nuclei were counterstained with DAPI and slides were mounted in mowiol. For image acquisition, an Imager Z.1 microscope (Zeiss, Germany) was used.

### Immunohistochemistry

Sections were immersed with PBS, permeabilized for 5 min with 0.1% triton/PBS, and blocked for 90 min with 5% goat serum at room temperature. Samples were incubated overnight at 4 °C with primary mouse α-PSMA (Abcam, UK, 1:250) and rabbit α-CD31 (Invitrogen, USA/MA, 1:100), or rabbit α-HIF1α (Novus biologicals, USA/CO, 1:100) antibodies. After washing 3 × with PBS, sections were incubated with goat α-mouse Alexa Fluor 488 (Thermo Fisher Scientific, USA/MA, 1:1000) and goat α-rabbit Alexa Fluor 555 (Cell Signaling, USA/MA, 1:1000) antibodies for 1 h at room temperature. DAPI (Merck, Germany) staining was applied for 3 min and the cells were mounted with mowiol. Images were acquired with an Imager Z.1 microscope (Zeiss, Germany).

### H&E staining

All following steps were performed at room temperature. Sections were immersed with PBS and rinsed with running water followed by incubation in Haematoxylin (10 min). Sections were rinsed again with running water and subsequently incubated in Eosin (1 min) followed by washing twice with running water for 5 min. Finally, dehydration steps (70% ethanol: 1 min, 96% ethanol: 1 min, 100% ethanol: 1 and 10 min, xylol: 1 and 10 min) were performed and tissues were mounted in pertex (Medite, Germany).

### Microautoradiography (mAURA) analysis of [^177^Lu]Lu-PSMA-I&T distribution

After the complete decay of injected ^177^Lu, tumor cryosections were washed 3 × with PBS and were incubated with 2 MBq [^177^Lu]Lu-PSMA-I&T per tissue for 4 h at room temperature. After washing, the slides were coated with NTB solution (Kodak, USA/NY) and incubated at − 20 °C in the dark. After 6 days the slides were equilibrated for 30 min at room temperature and incubated in developer (Tetenal, Germany) (5 min), destilled water (0.5 min), fixing solution (Tetenal, Germany) (10 min), and destilled water (10 min) and stained with H&E.

### Autoradiography (AURA) analysis of [^177^Lu]Lu-PSMA-I&T distribution

Tumor cryosections were washed with PBS and incubated with 2 MBq [^177^Lu]Lu-PSMA-I&T per tissue for 4 h at RT. After washing, sections were exposed on plates (Fuji Film BAS-IP SR 2025, Raytest, Germany) for 24 h at room temperature and scanned using the Typhoon FLA 7000 (GE Healthcare, USA/IL) to obtain an image of the [^177^Lu]Lu-PSMA-I&T distribution.

### Statistical analysis

All statistical analyses were performed using the Graph-Pad Prism software (Version 8). Data are expressed as mean ± SD. Kaplan–Meier survival curve was analyzed with the log-rank test. Comparison of mean values was analyzed with ANOVA and Tukey’s post-hoc correction after testing for normal distribution.

## Results

### [^177^Lu]Lu-PSMA-I&T decelerates tumor progression and increases survival rates in TNBC xenografted mice

**Fig. 1:**
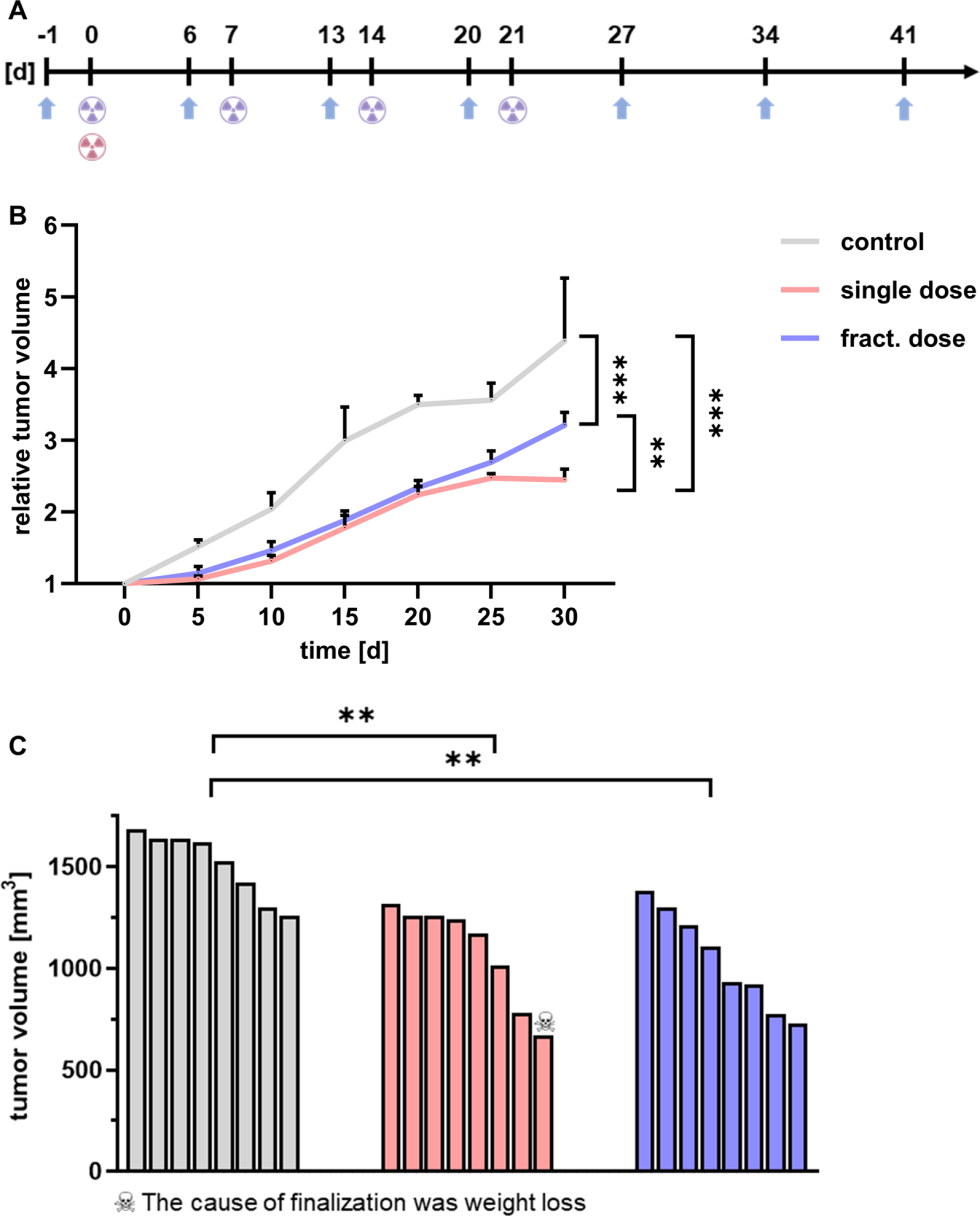
**A** Schematic therapy regime of single dose (pink) and fractionated dose (violet) of [^177^Lu]Lu-PSMA-I&T. Tumor growth was monitored weekly via [^18^F]FDG-PET/CT (blue arrows). Day 0 marks the start of the treatment. **B** Tumor growth curve in control (gray), single dose (pink) and fractionated dose therapy (violet) animals (n ≥ 5); ** *p* = 0.002, *** *p* < 0.001). **C** Waterfall plot analysis of tumor volumes on the day of finalization. Volumetric differences were significant for the single dose (** *p* = 0.003) and fractionated dose (** *p* = 0.001) treated animals when compared to the control group

The therapeutic effect of [^177^Lu]Lu-PSMA-I&T was analyzed after intravenous administration of a single dose (60 MBq) or four fractionated doses every 7 days (4 × 15 MBq) (Fig. 1A). Tumor growth was monitored using [^18^F]FDG-PET/CT. The tumor volume 30 days after therapy start was significantly smaller for the single dose (*p* < 0.001) and fractionated dose (*p* < 0.001) groups compared to the control (Fig. 1B). Tumor growth analysis ended on day of finalization of the last control animal. Interestingly, only control group animals reached the end point for tumor volume, while the remaining animals were finalized solely due to tumor diameter. One single dose animal was finalized due to weight loss, however no specific systemic radiotoxicity was detected. The mean calculated tumor volumes on the day of finalization were 1511, 1088, and 1044 mm^3^ for control, single dose, and fractionated dose therapy groups respectively (Fig. 1C). Statistical analysis revealed significantly smaller tumor volumes at the time of finalization of the single dose (*p* = 0.003) and fractionated dose (*p* = 0.001) treated animals compared to the control.

**Fig. 2:**
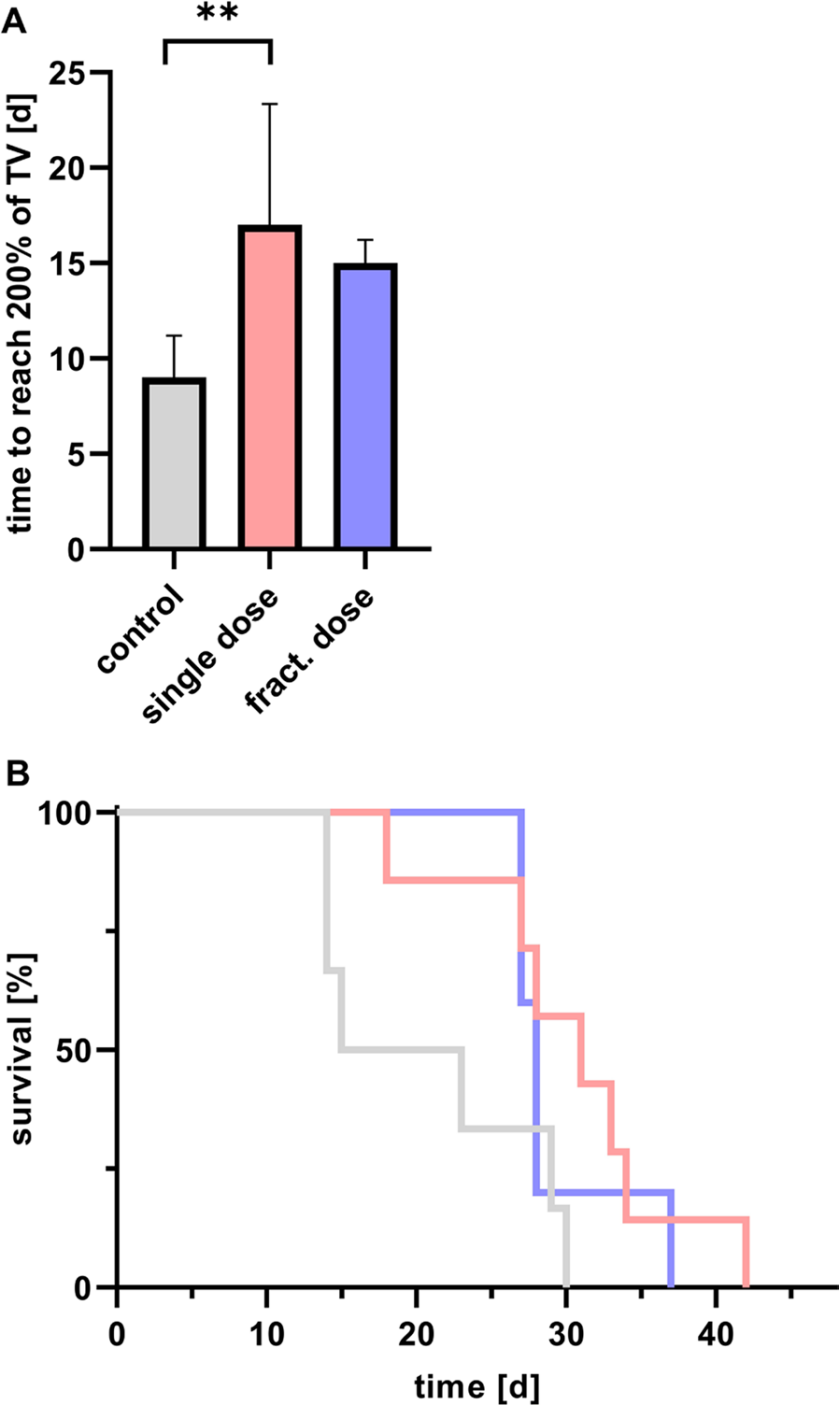
**A** Tumor growth analysis comparing the time period (in days) until reaching 200% of the initial tumor volume (TV) in control and therapy receiving groups (n ≥ 5); ** *p* = 0.008). **B** Kaplan–Meier survival of single dose and fractionated dose group compared to the control

Moreover, tumor growth was clearly inhibited in both treatment groups, as the control group reached 200% of the initial tumor volume faster than the single dose or fractionated dose animals (9 d *vs.* 17 d *vs.* 15 d, respectively) (Fig. 2A). The calculated relative tumor growth inhibition rates were 38% and 30% for single dose and fractionated dose therapy. The Kaplan–Meier anaylsis indicated a median survival of 19 d for the control group, 31 d for the single dose, and 28 d for the fractionated dose group (Fig. 2B). However, the increased survival rates after therapy did not reach statistical significance.

### [^18^F]FDG tumor uptake decreases after [^177^Lu]Lu-PSMA-I&T treatment

**Fig. 3:**
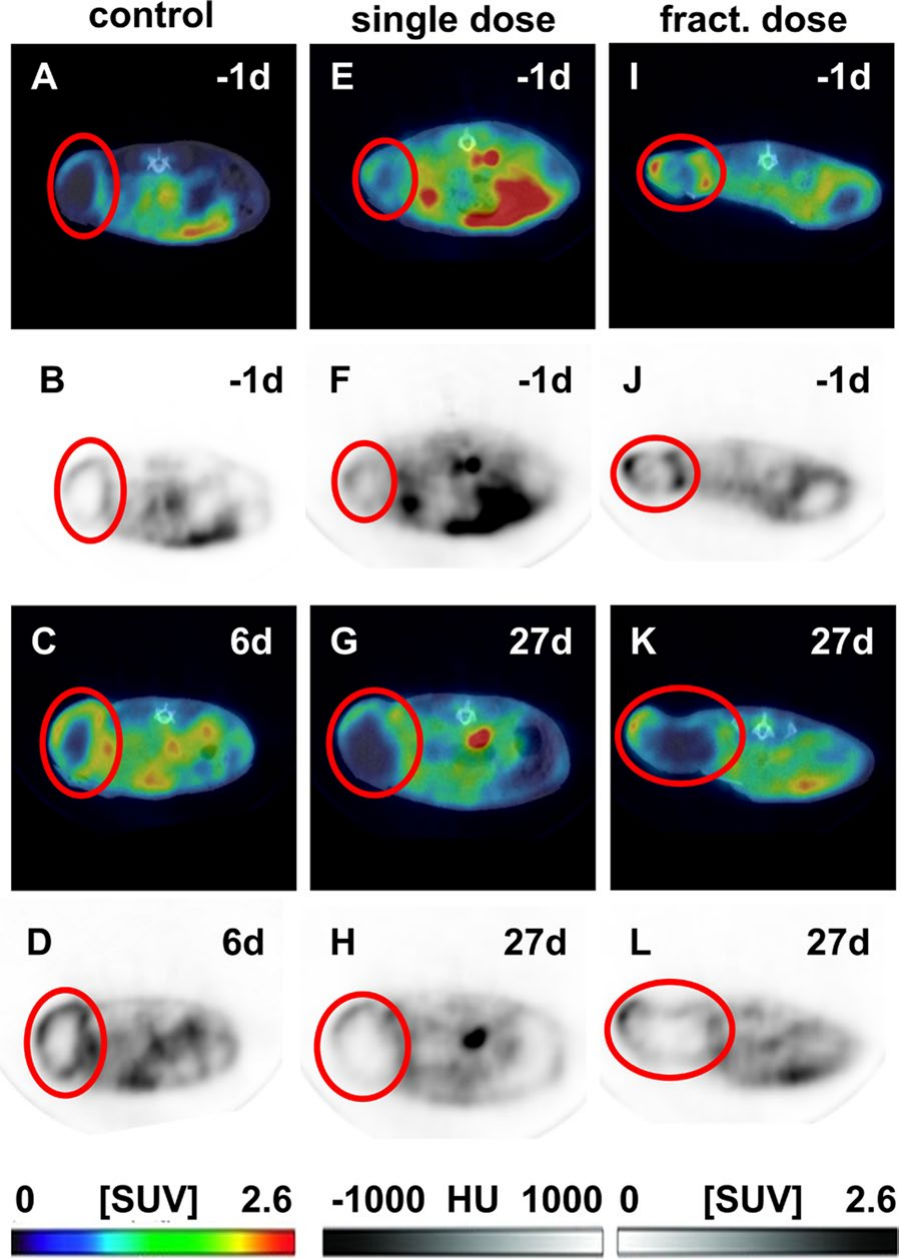
Representative PET/CT images of [^18^F]FDG distribution 30 min post injection in control (**A**–**D**), single dose (**E**–**H**), and fractionated dose (**I**–**L**) group before (− 1d) and 6 or 27 d after therapy. Colored images represent PET/CT images with a SUV_max_ of 2.6 and HU range from − 1000 to 1000. Black and white images represent PET with a SUV_max_ of 2.6

The tumor growth was monitored via weekly [^18^F]FDG-PET/CT performed at 30 min post injection (Fig. 3, Additional file 1: Figs. S1–S6). Due to the fast tumor growth and defined finalization criteria the latest monitoring time point in the control group was day 6 after initiaton of the therapy study. The control group showed a clear increase in tumor uptake on day 6 compared to -1d. For both therapy groups, the [^18^F]FDG uptake decreases despite increased tumor volume as indicated at day 27 post treatment.

### [^177^Lu]Lu-PSMA-I&T induces apoptosis in TNBC tumors

**Fig. 4:**
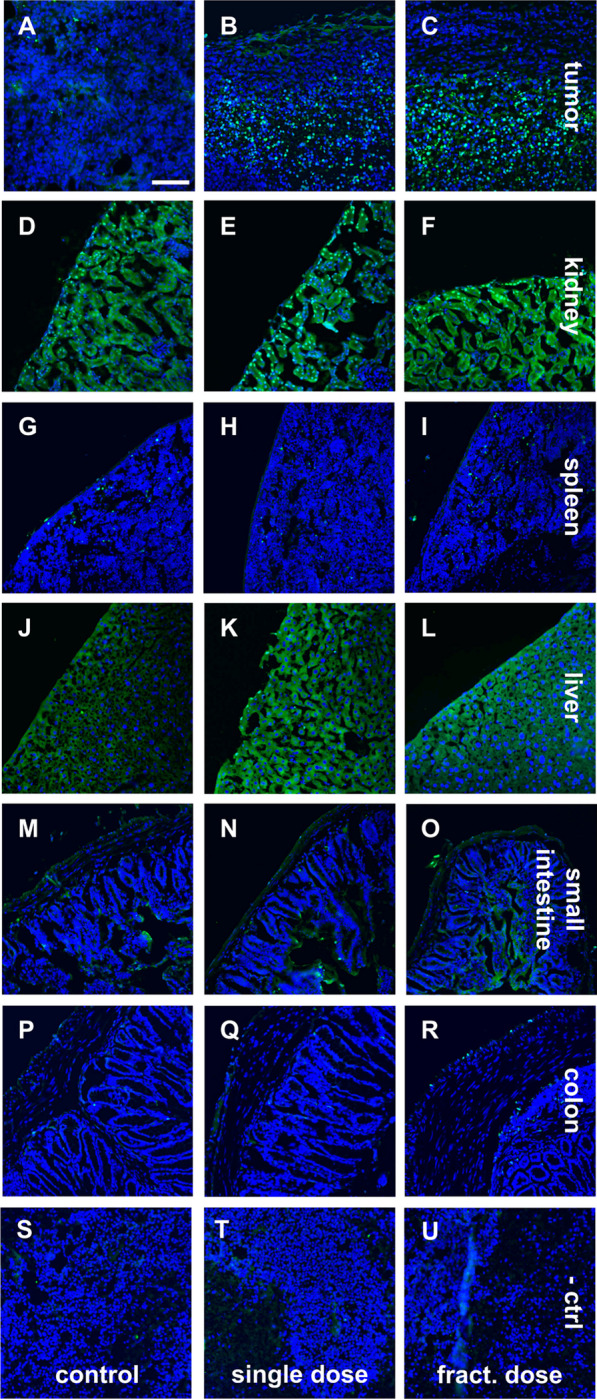
TUNEL staining (green) of tumor and organs of control (**A**, **D**, **G**, **J**, **M**, **P**), single dose (**B**, **E**, **H**, **K**, **N**, **R**, **Q**) and fractionated dose group (**C**, **F**, **I**, **L**, **O**, **R**). Nuclei were counterstained with DAPI (blue). The staining solution without enzyme served as negative control (**S**–**U**). Scale bar: 100 µm

The apoptotic effect in isolated organs was visualized using ex vivo TUNEL staining (Fig. 4). In the tumor tissue, both therapy groups showed a higher amount of apoptotic cells compared to the control animals. Importantly, this effect was observed exclusively at the tumor edges (Fig. 4A–C). The core region of the tumors was highly apoptotic/necrotic in all groups (Additional file 1: Fig. S7). To evaluate the apoptotic off-side effects, kidney, spleen, liver, small intestine, and colon were also analyzed. The kidney was the only organ which showed some single apoptotic cells. However, these are not caused by the radiation as they also appear in the control group. The other organs revealed no apoptosis in therapy or control groups. Additional H&E staining revealed no morphological changes induced by the treatment (Additional file 1: Fig. S8).

### Treatment with [^177^Lu]Lu-PSMA-I&T increases intratumoral hypoxia

**Fig. 5:**
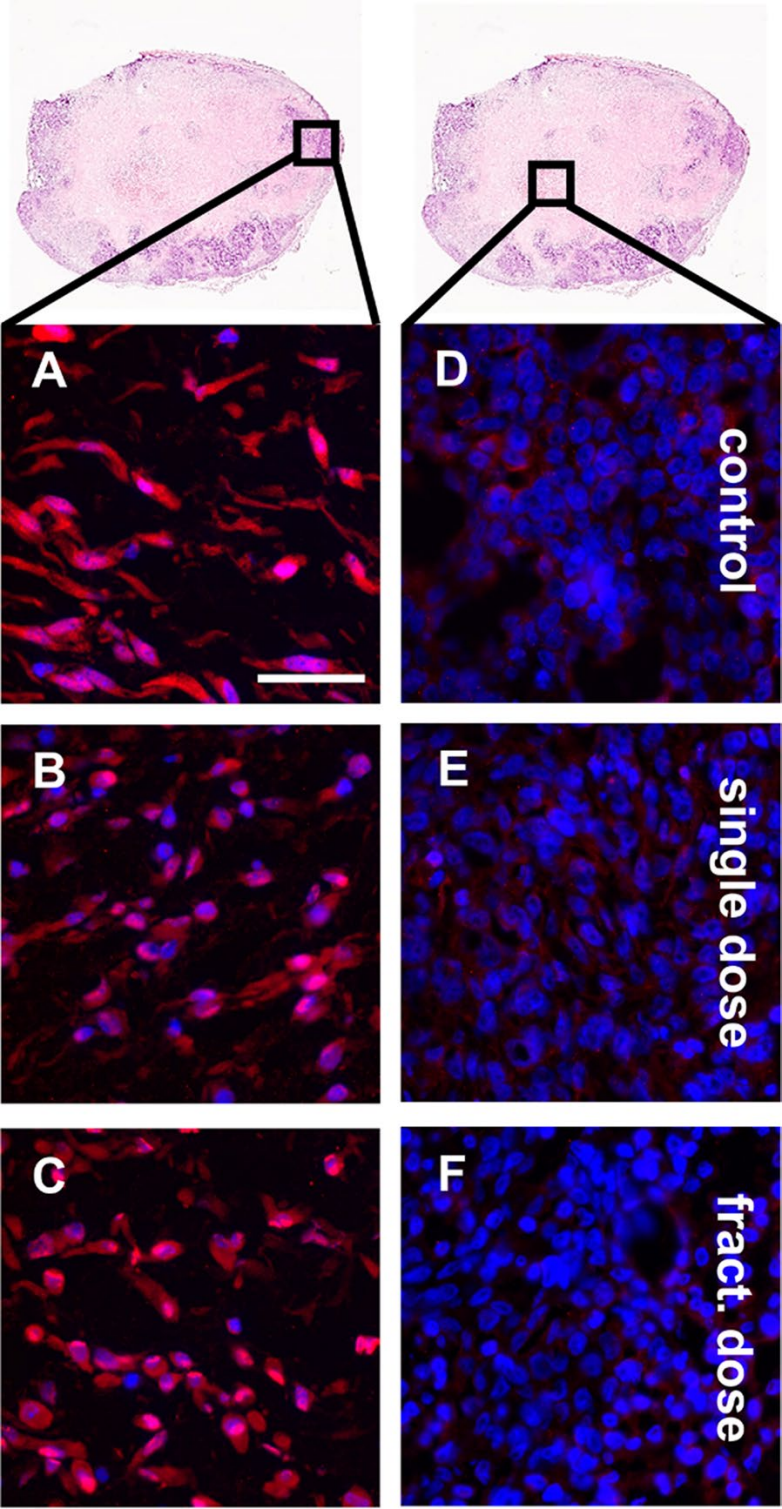
Microscopic evaluation of HIF1α (red) expression in tumor tissue from control (**A**, **D**), single dose therapy (**B**, **E**), and fractionated dose therapy (**C**, **F**) animals. Nuclei were counterstained with DAPI (blue). The black box inside the schematic tumor tissue illustrates the location of interest. Scale bar: 50 µm

For further therapy evaluation, tumor tissue was stained with α-HIF1α antibody (red) (Fig. 5). In the tumor edge regions of control animals, the hypoxia marker HIF1α was detected in the nucleus and cytoplasm (Fig. 5a). Unlike in the therapy animals, where HIF1α is preferentially located in the cell nucleus (Fig. 5B, C). The tumor core in all study groups was negative for HIF1α (Fig. 5D–F).

### [^177^Lu]Lu-PSMA-I&T binds to tumor-associated vasculature in TNBC

**Fig. 6:**
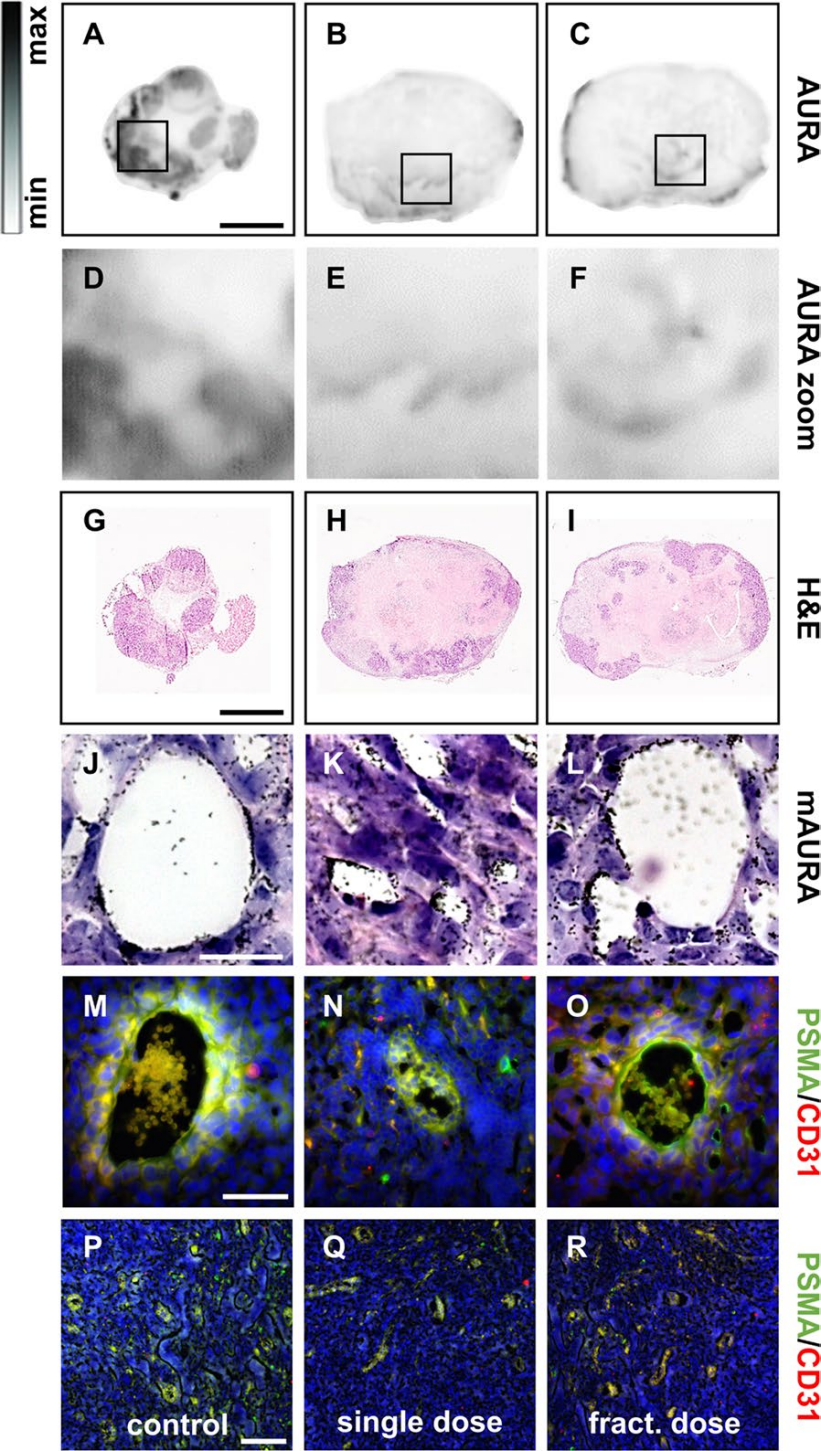
Evaluation of PSMA expression in TNBC tumors using [^177^Lu]Lu-PSMA-I&T AURA (**A**–**C**; zoom-in: **D**–**F**) with corresponding H&E staining (**G**–**I**); and [^177^Lu]Lu-PSMA-I&T mAURA (**J**–**L**) with corresponding PSMA/CD31 immunofluorescence staining analysis (**M**–**R**). Scale bars: **A**–**C** & **G**–**I**: 4 mm, **J**–**L**: 20 µm; **M**–**O**: 50 µm; **P**–**R**: 100 µm

The binding of [^177^Lu]Lu-PSMA-I&T was verified via AURA, mAURA, and immunostaining of tumor tissue sections (Fig. 6). The control tumor demonstrates tremendously higher [^177^Lu]Lu-PSMA-I&T binding compared to the therapy groups (Fig. 6A–F). A corresponding H&E staining is included for reference (Fig. 6G–I). Noteworthy, tumors of the therapy groups show more necrotic tissue (light pink) and less viable areas compared to the control. The mAURA with [^177^Lu]Lu-PSMA-I&T demonstrates clear accumulation of the silver grains on the walls of the blood vessels in all groups (Fig. 6J–L). Tumor tissues were co-stained with α-PSMA and α-CD31 to prove PSMA expression in the blood vessels (Fig. 6M–R; Additional file 1: Figs. S9 + S10). Secondary antibody controls were used to exclude unspecific staining (Additional file 1: Fig. S11).

## Discussion

Based on previous findings indicating the expression of PSMA in tumor-associated endothelial cells in TNBC [11–13] and in the tumor vasculature of TNBC patient samples [10], we analyzed the therapeutic efficacy of [^177^Lu]Lu-PSMA-I&T endogenous radiotherapy in orthotopic TNBC xenografts in a single and a fractionated dose approach. Analyzing tumor volume, tumor growth inhibition, and overall survival of the mice (Figs. 1, 2) we could demonstrate the efficacy of both therapeutic strategies. Considering the inhibition of the tumor growth the single cycle therapy was even more effective compared to the fractionated approach (*p* = 0.002). For low-dose radiotherapies, iterative re-oxygenation of tumor tissue has been described. With this approach, the blood vessels stay intact whereas a single high-dose irradiation effectively damages the tumor-associated vasculature [18]. This could be a reasonable explanation for the observation that the single dose treatment with [^177^Lu]Lu-PSMA-I&T more efficiently inhibited the tumor growth than the fractionated dose. Moreover, the fraction of 15 MBq might be not sufficient to optimally arrest tumor growth. Future dose escalating studies will be needed to disentangle the necessary amount of the administered doses to receive the most efficient tumor growth inhibiting effect of [^177^Lu]Lu-PSMA-I&T. In the evaluated xenograft model, the PSMA expression on the TNBC cells itself was rather low, indicating that a direct uptake of the radiopharmaceutical from the tumor cells is not necessary to inhibit the growth of the tumor. Targeting the tumor-associated vasculature therefore demonstrates an efficient approach and additionally, the cross-fire effect may contribute to the efficacy by ensuring that the irradiation reaches PSMA-low expressing tumor cells in direct proximity to the PSMA-high expressing endothelial cells counteracting a certain degree of heterogeneous radionuclide distribution [19].

The tumor growth was monitored with [^18^F]FDG-PET/CT (Fig. 3). On the sequential PET/CT scans differences regarding the intratumoral uptake of [^18^F]FDG over time was indicated with a clear decrease in the therapy groups, and an increase in the control animals. A decrease in [^18^F]FDG uptake indicates a reduction of vital tumor tissue areas, therefore the SUV_mean_ is also used as a measure for therapy response. Additionally, high [^18^F]FDG uptake indicates abnormally high metabolic rates in tumors and often correlates with aggressiveness of the disease [20]. No [^18^F]FDG uptake was detected in the core region of the tumor due to the spontaneous central necrosis formation, which was demonstrated for MDA-MB-231 xenografts in several studies before [21, 22]. However, despite the fact that the necrotic core contributes to the overall tumor volume, the vital tumor tissue (indicated by [^18^F]FDG uptake) decreased under treatment with [^177^Lu]Lu-PSMA-I&T.

The induced apoptotic effect of [^177^Lu]Lu-PSMA-I&T on the tumor itself as well as in several organs was evaluated with the TUNEL assay (Fig. 4). Due to the necrotic tumor core present in all study groups, the therapeutic effect was assessed exclusively in the edge tumor areas. Here, the therapy groups showed more apoptosis than the control group. Besides of kidneys, no apoptosis was detected in any of the analyzed organs underlining the safety of [^177^Lu]Lu-PSMA-I&T in this setting. The minor apoptotic effect in kidneys is probably due to the murine-specific expression of PSMA in renal tubules and Bowman's capsule [23]. Similarly, in PCa patients, both [^177^Lu]Lu-PSMA-I&T and [^177^Lu]Lu-PSMA-617 are reported to be safe and well tolerated with only minor and easily manageable side effects [24, 25].

Hypoxia plays a major role in cancer progression and is often a predictor for therapy resistance and poor prognosis. Oxygen-deprived cancer cells are more prone to survive radiation therapy than normoxic cells, therefore a higher dose is needed to achieve the same therapeutic effect [18]. For the untreated tumor, the microscopic examination of HIF1α expression revealed its cytosolic and nuclear localization (Fig. 5). Interestingly, after treatment with [^177^Lu]Lu-PSMA-I&T HIF1α signal was predominantly detected in the nucleus. A similar effect was shown for external radiotherapy in TNBC cells [26]. As indicated by mAURA and AURA of tumor tissue sections, [^177^Lu]Lu-PSMA-I&T targeted the tumor-associated vasculature leading to a decrease in oxygen supply to the tumor cells. In response to the hypoxic environment, HIF1α translocates from the cytosol to the nucleus and there, it interacts with HIF1β [27]. However, hypoxia triggers neoangiogenesis through the upregulation of different pro-angiogenic factors including VEGF [28]. Further it promotes the production of reactive oxygen species [29]. TNBC cells adapt very well to the hypoxic state by increasing their intracellular concentration of the anti-oxidant glutathione [30]. Glutathione consists of glycine, cysteine, and glutamate. PSMA as a glutamate carboxidase produces glutamate, which in turn can be used to generate glutathione, resulting in increased resistance of the cells against oxidative stress [31].

Moreover, a decrease in PSMA after [^177^Lu]Lu-PSMA-I&T treatment was observed with autoradiographic analysis (Fig. 6). As demonstrated in prostate cancer patients, therapy with [^177^Lu]Lu-PSMA-I&T decreased prostate-specific antigen (PSA) levels by at least 25% after 4 weeks [32]. A different study found, that total lesion-PSMA positively correlates both with Gleason score and PSA levels [33] assuming that a decrease in PSA levels is accompanied by a decrease in PSMA levels. The corresponding H&E images further demonstrate, that the tumor tissue of the treated groups contains less viable regions compared to the control group, assuming that [^177^Lu]Lu-PSMA-I&T efficiently decreased the blood supply and thus the overall cell viability. Further we could prove the presence of PSMA on the tumor-associated blood vessels via mAURA and immunostaining, verifying the expectation that the tumor growth inhibition is a direct consequence to the irradiation of the tumor-associated vasculature.

Angiogenesis inhibitors are already implied in the treatment of breast cancer. The direct inhibitors prevent the proliferation of endothelial cells (e.g. angiostatin), whereas indirect inhibitors bind to pro-angiogenic factors or their respective receptors and block their activity (e.g. bevacizumab: neutralization of VEGF) [34]. However, despite progressing research, most angiogenesis inhibitors still fail to significantly improve patient survival. In the past years, the utilization of radiolabeled anti-angiogenic agents has emerged as an attractive strategy. The advantage over non-radioactive agents is the ability to target surrounding tumor cells, induced by the crossfire effect and the radiation-bystander effect [35].

These radiation effects might contribute to the therapeutic potential of [^177^Lu]Lu-PSMA-I&T. Considering the predominant PSMA expression on the vasculature, [^177^Lu]Lu-PSMA-I&T destroys the tumor-associated endothelial cells and therefore stops or decreases the blood supply to the tumor.

## Conclusion

The prognosis of patients diagnosed with TNBC is poor and due to the lack of therapeutic options, targeted strategies are urgently searched for. To our knowledge, this is the first in vivo evaluation of [^177^Lu]Lu-PSMA-I&T therapy in TNBC mice comparing a single dose and a fractionated dose approach. It is clearly demonstrated that [^177^Lu]Lu-PSMA-I&T inhibits tumor growth and improves survival. [^177^Lu]Lu-PSMA-I&T accumulates in the blood vessels and thus acts as an anti-angiogenic radio therapeutics. Further studies need to address the dosimetric aspect to improve the therapeutic effect of the fractionated dose. Moreover, it would be interesting to test if a combination of [^177^Lu]Lu-PSMA-I&T and chemotherapy could enhance the tumor growth inhibiting effect.

## Limitations of the study

We are aware that experiments regarding biodistribution and toxicity are not included in this study. [^117^Lu]Lu-PSMA-I&T is a very common and frequently used radiotracer, therefore the biodistribution and toxicity were already sufficiently evaluated before. Especially regarding the amount of animals, one should use as much as necessary and as little as possible. In this first in vivo attempt using [^177^Lu]Lu-PSMA-I&T in TNBC, we used relatively small groups of n = 8 mice per group. After demonstrating efficacy of the treatment, the n-number can be increased for further optimization of the therapy in future studies. Also, we propose to perform a dosimetry study to calculate the optimal injected dose. Another limitation is the high amount of necrosis formation in the MDA-MB-231 xenograft, which is characteristic for this cell line, assuming that the vascularization of the tumor is moderate. To address this, a combinational approach with [^117^Lu]Lu-PSMA-I&T and chemotherapeutics could be useful to increase the efficacy of the treatment.

### Supplementary Information

**Additional file 1.** Supplementary information. Supplementary figures S1–S11.

## Data Availability

All data generated or analyzed during this study are available from the corresponding author.

## Abbreviations

AURA: Autoradiography
HUVECs: Human umbilical vein endothelial cells
mAURA: Microautoradiography
PSA: Prostate-specific antigen
PSMA: Prostate-specific membrane antigen
TNBC: Triple-negative breast cancer
VEGF: Vascular endothelial growth factor
