# Supplementary material for: The prostate-specific membrane antigen holds potential as a vascular target for endogenous radiotherapy with [177Lu]Lu-PSMA-I&T for triple-negative breast cancer
Source: Breast Cancer Res. 2024 Feb 20;26:30. doi: 10.1186/s13058-024-01787-9 (PMC10877802; doi:10.1186/s13058-024-01787-9)
Supplement: Supplementary file 1 — Additional file 1. Supplementary information. Supplementary figures S1–S11. [file 13058_2024_1787_MOESM1_ESM.docx]

***Supplementary information***

***Results***

**
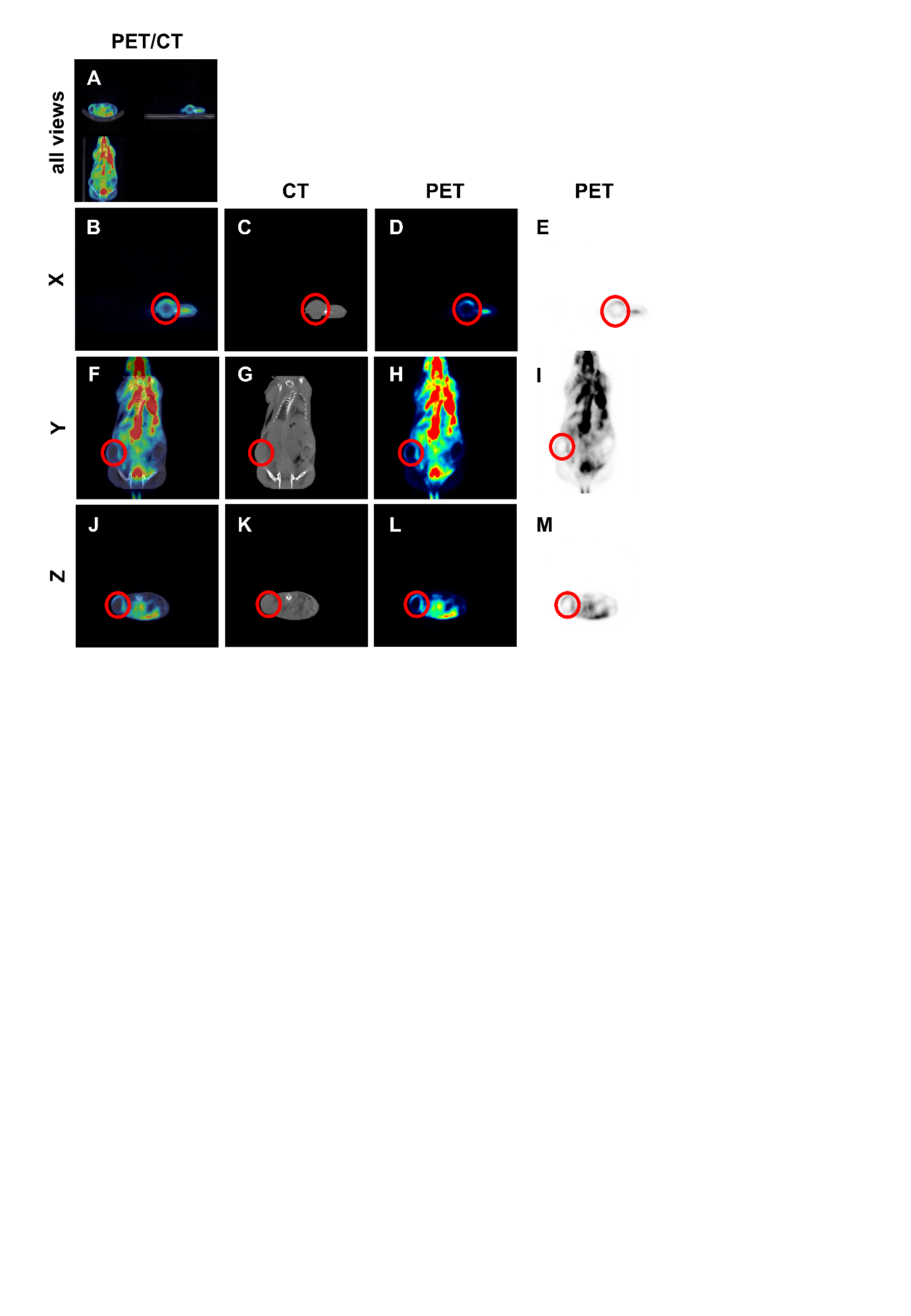
**

Figure S1. Representative PET/CT (A, B, F, J) images of [^18^F]FDG distribution 30 min post injection in the control animal before (-1d) therapy fused from the CT (C, G, K) and PET images (D, E, H, I, L, M). Animals are presented in sagittal (X), coronal (Y), and axial (Z) plane. Scales: SUV 0-2.6; HU -1000-1000.

**
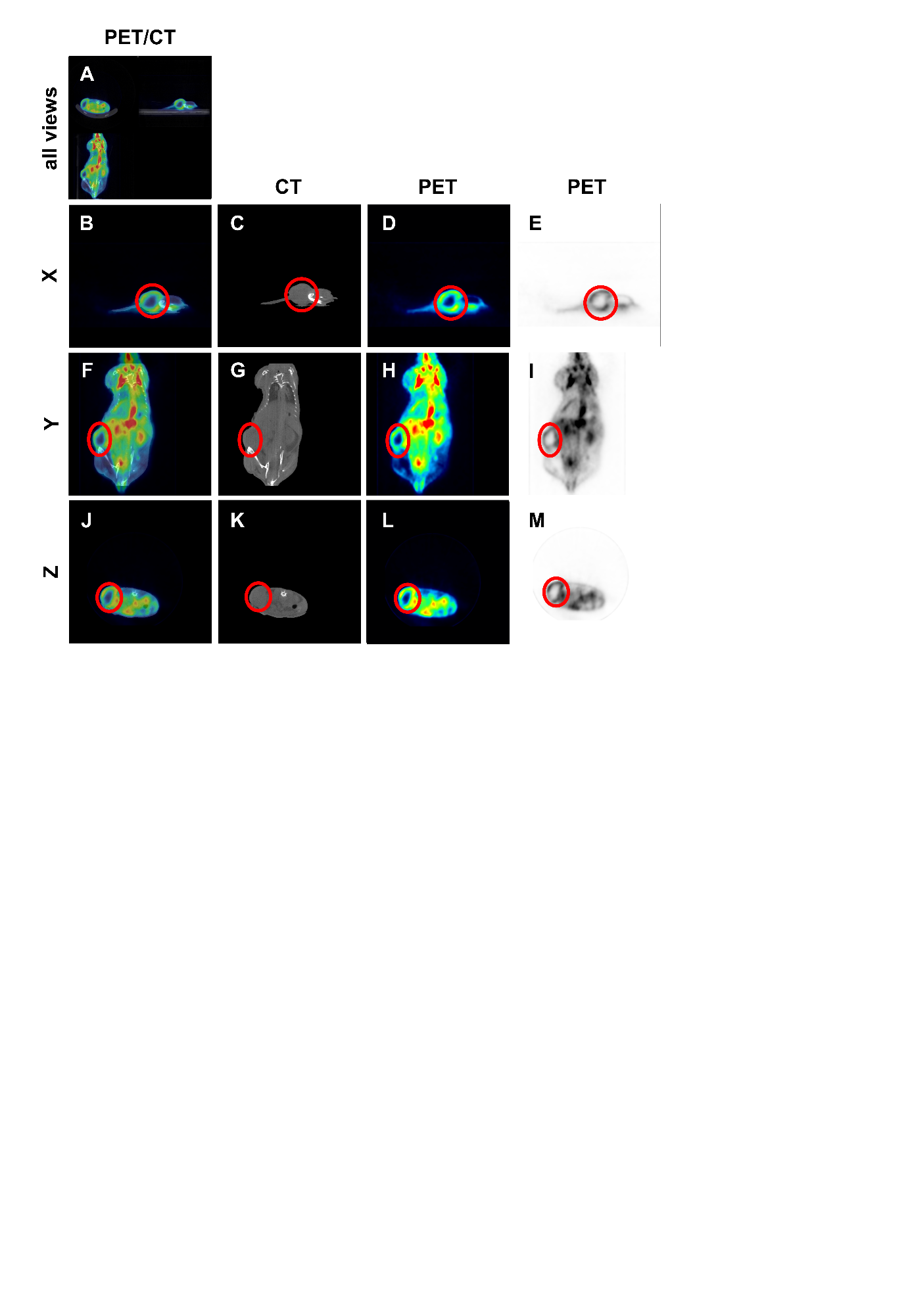
**

Figure S2. Representative PET/CT (A, B, F, J) images of [^18^F]FDG distribution 30 min post injection in the control animal 6 d after therapy fused from the CT (C, G, K) and PET images (D, E, H, I, L, M). Animals are presented in sagittal (X), coronal (Y), and axial (Z) plane. Scales: SUV 0-2.6; HU -1000-1000.

**
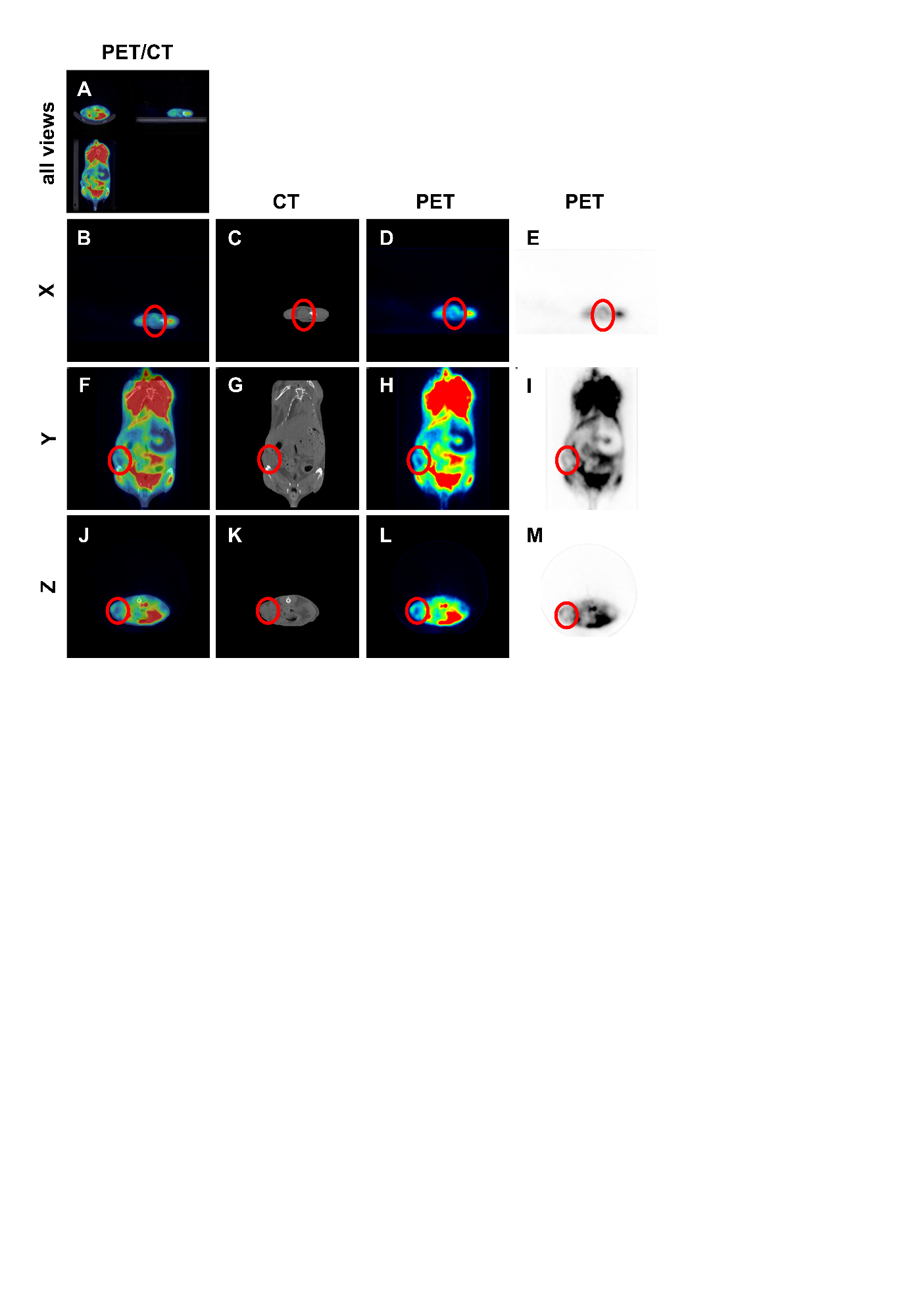
**

Figure S3. Representative PET/CT (A, B, F, J) images of [^18^F]FDG distribution 30 min post injection before (-1d) therapy in the single dose treatment animal fused from the CT (C, G, K) and PET images (D, E, H, I, L, M). Animals are presented in sagittal (X), coronal (Y), and axial (Z) plane. Scales: SUV 0-2.6; HU -1000-1000.

**
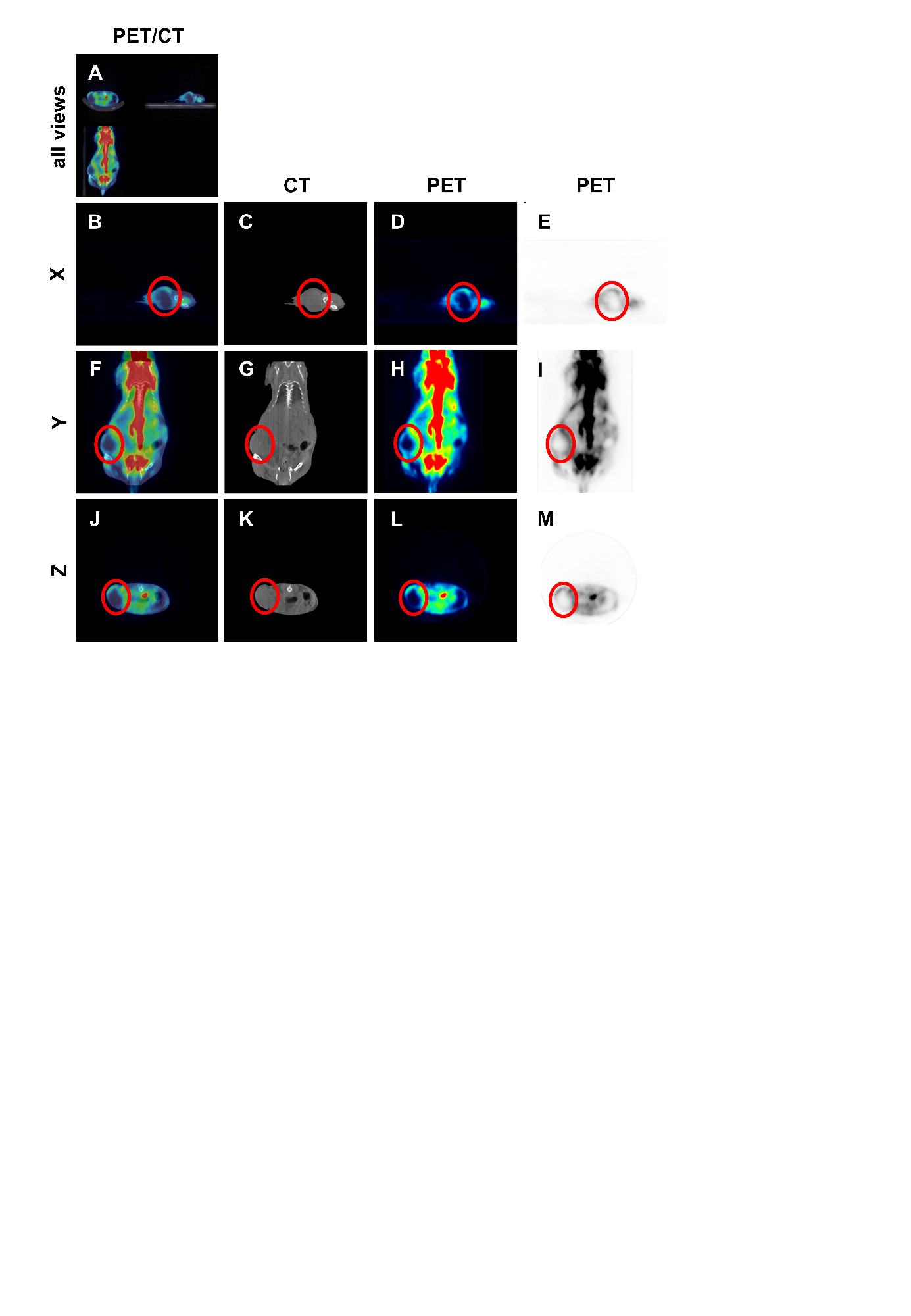
**

Figure S4. Representative PET/CT (A, B, F, J) images of [^18^F]FDG distribution 30 min post injection 27 d after therapy in the single dose treatment animal fused from the CT (C, G, K) and PET images (D, E, H, I, L, M). Animals are presented in sagittal (X), coronal (Y), and axial (Z) plane. Scales: SUV 0-2.6; HU -1000-1000.

**
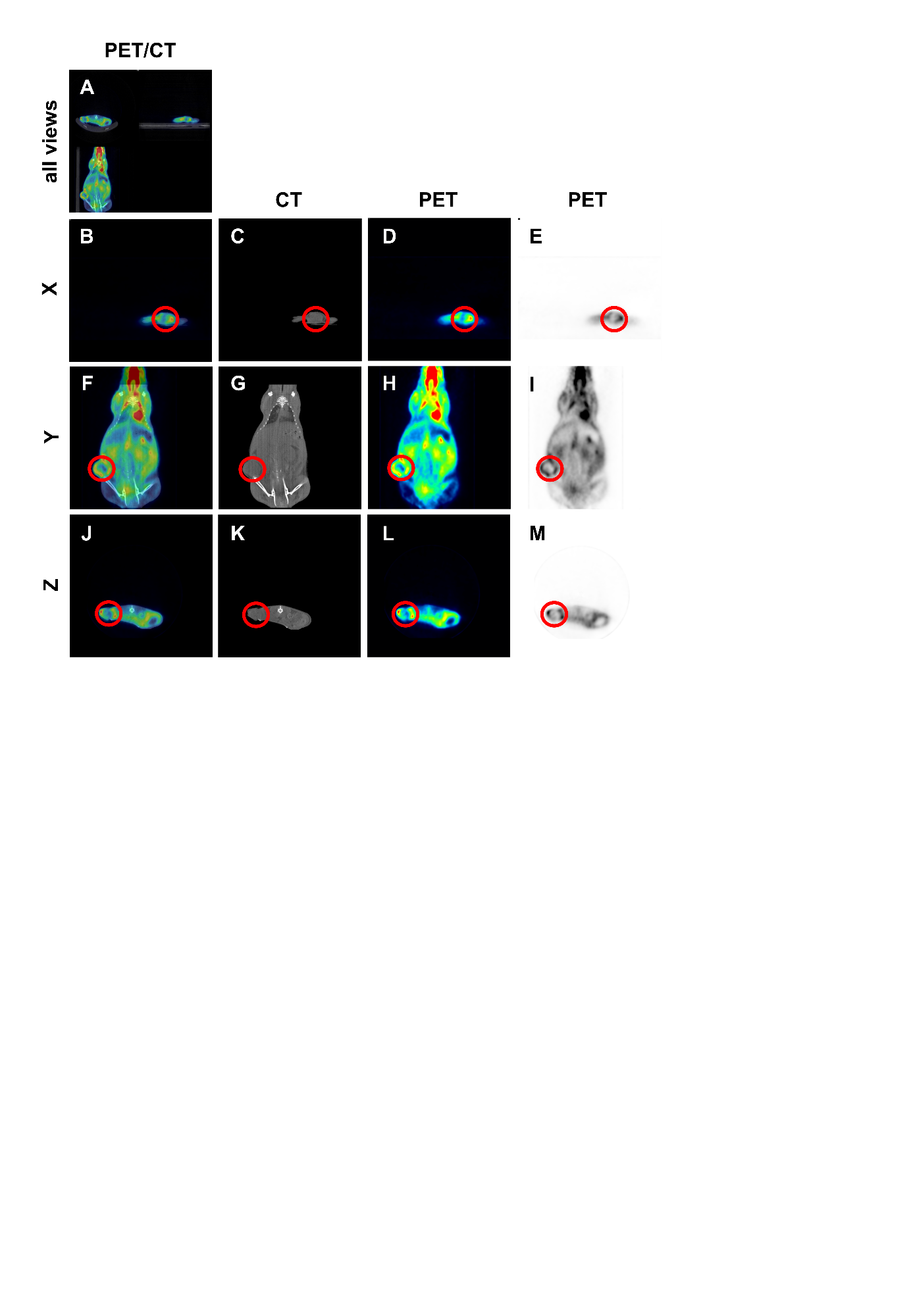
**

Figure S5. Representative PET/CT (A, B, F, J) images of [^18^F]FDG distribution 30 min post injection before (-1d) therapy in the fractionated dose treatment animal fused from the CT (C, G, K) and PET images (D, E, H, I, L, M). Animals are presented in sagittal (X), coronal (Y), and axial (Z) plane. Scales: SUV 0-2.6; HU -1000-1000.

**
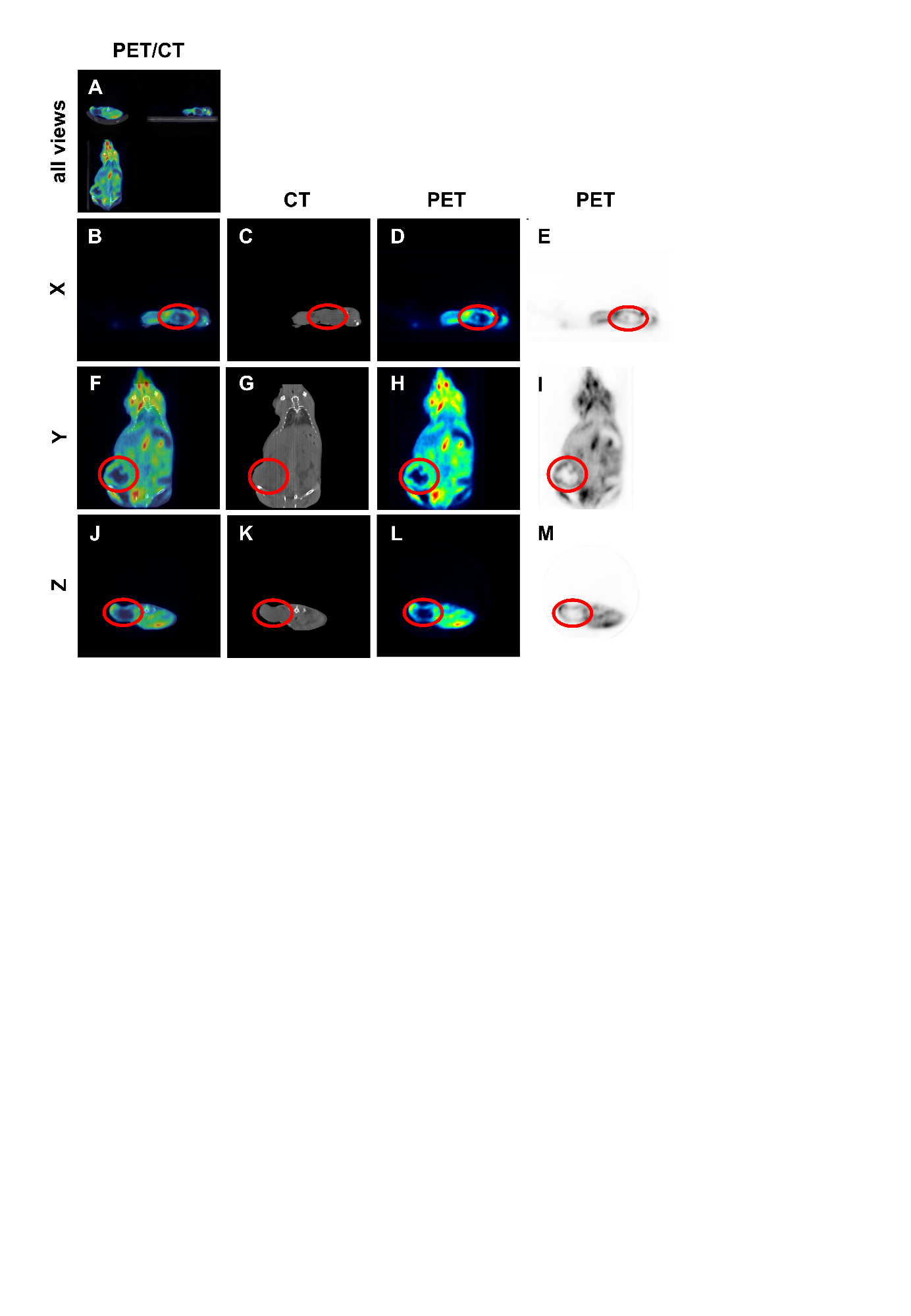
**

Figure S6. Representative PET/CT (A, B, F, J) images of [^18^F]FDG distribution 30 min post injection 27 d after therapy in the fractionated dose treatment animal fused from the CT (C, G, K) and PET images (D, E, H, I, L, M). Animals are presented in sagittal (X), coronal (Y), and axial (Z) plane. Scales: SUV 0-2.6; HU -1000-1000.


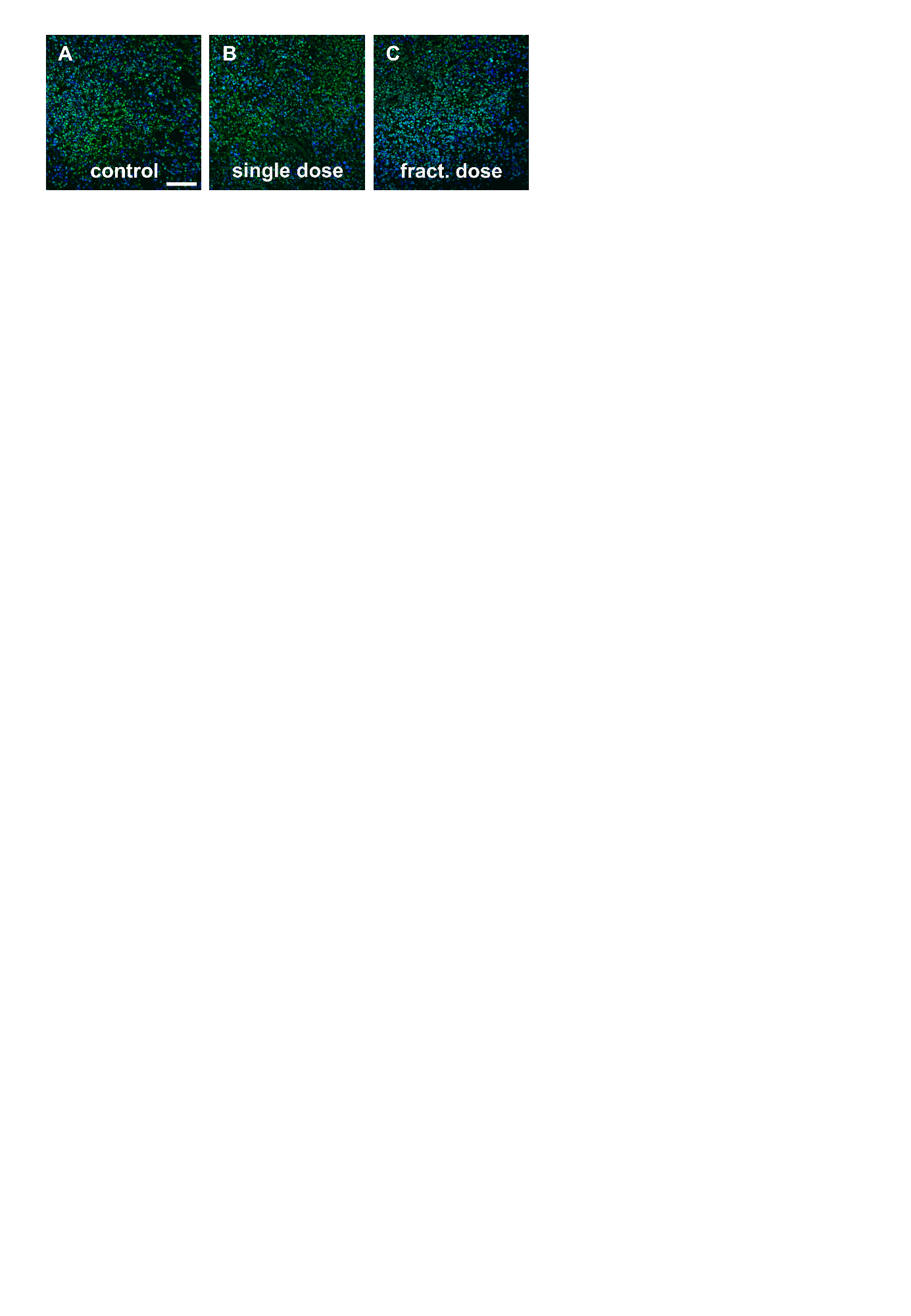


Figure S7. TUNEL staining (green) of the core region of the tumor of control (A), single dose (B) and fractionated dose (C) treated groups. Nuclei were counterstained with DAPI (blue). Scale bar: 100 µm.


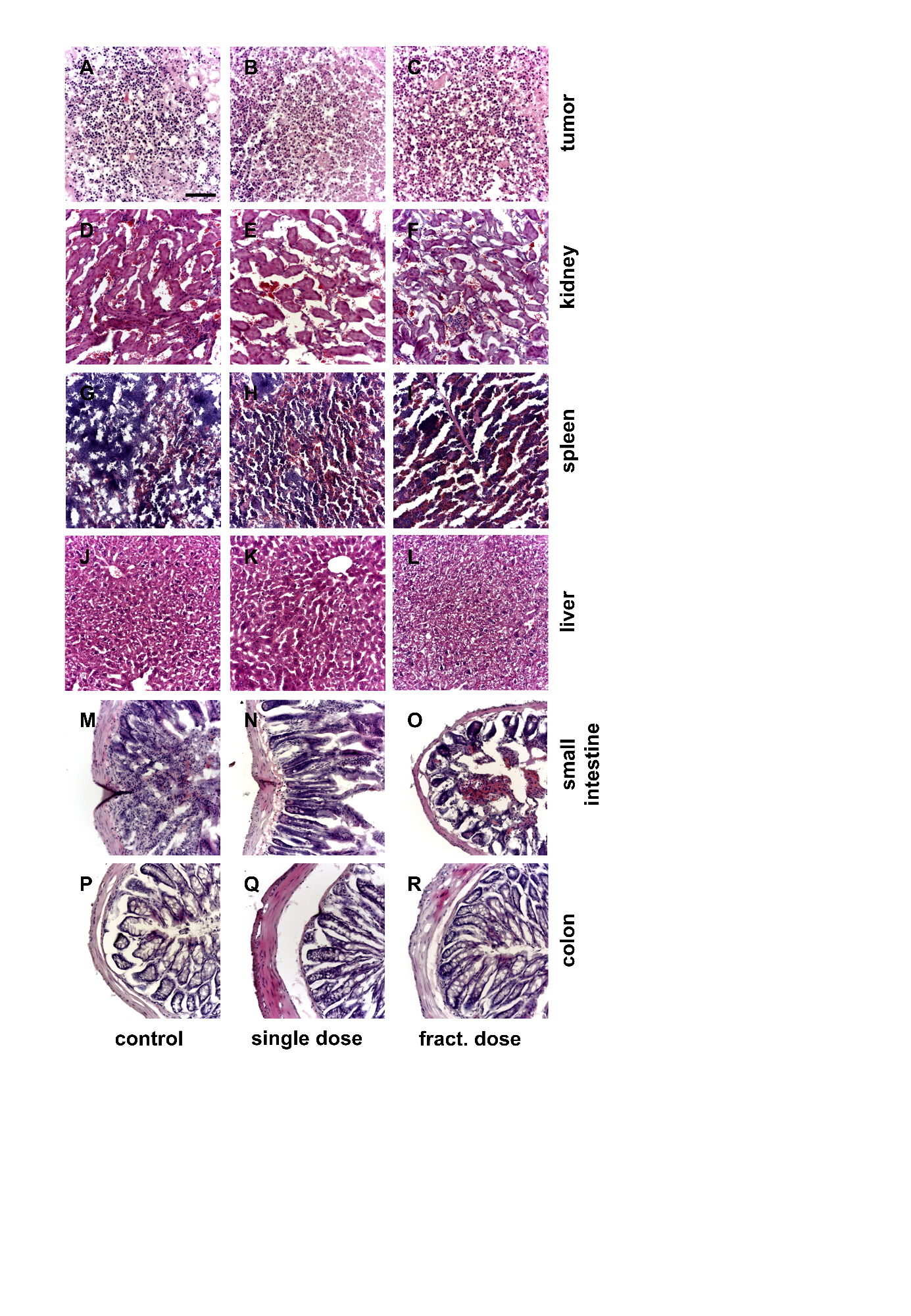


Figure S8. H&E staining of the organs isolated from control (A, D, G, J, M, P), single dose (B, E, H, K, N, Q) and fractionated dose (C, F, I, L, O, R) treated animals.

**
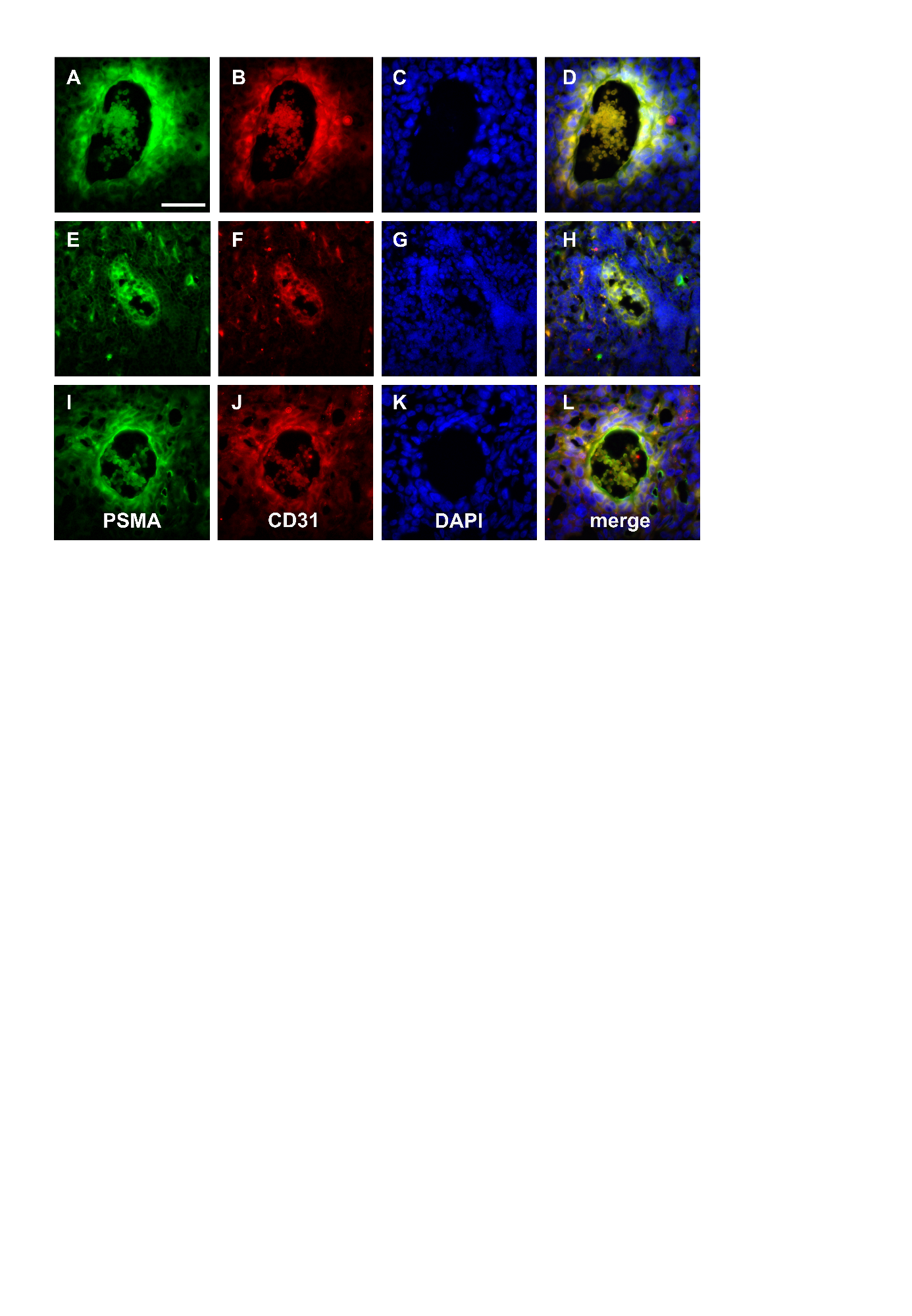
**

Figure S9. Single channels pictures of Fig. 6 M-O. α-PSMA (green) and α-CD31 (red) staining on tumor tissue was counterstained with DAPI (blue). Scale bar: 50 µm.


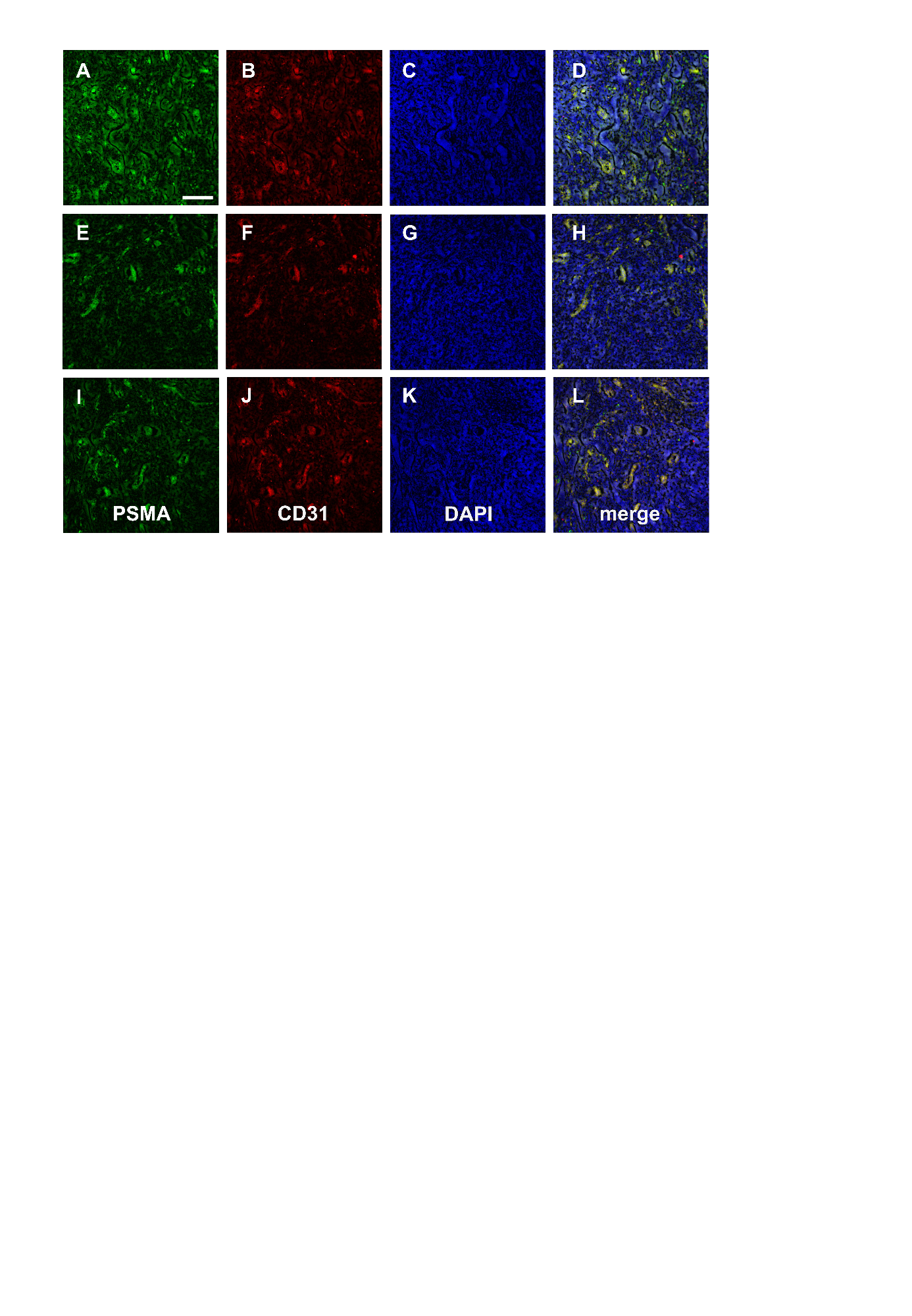


Figure S10. Single channels pictures of Fig. 6 P-R. α-PSMA (green) and α-CD31 (red) staining on tumor tissue was counterstained with DAPI (blue). Scale bar: 100 µm.

**
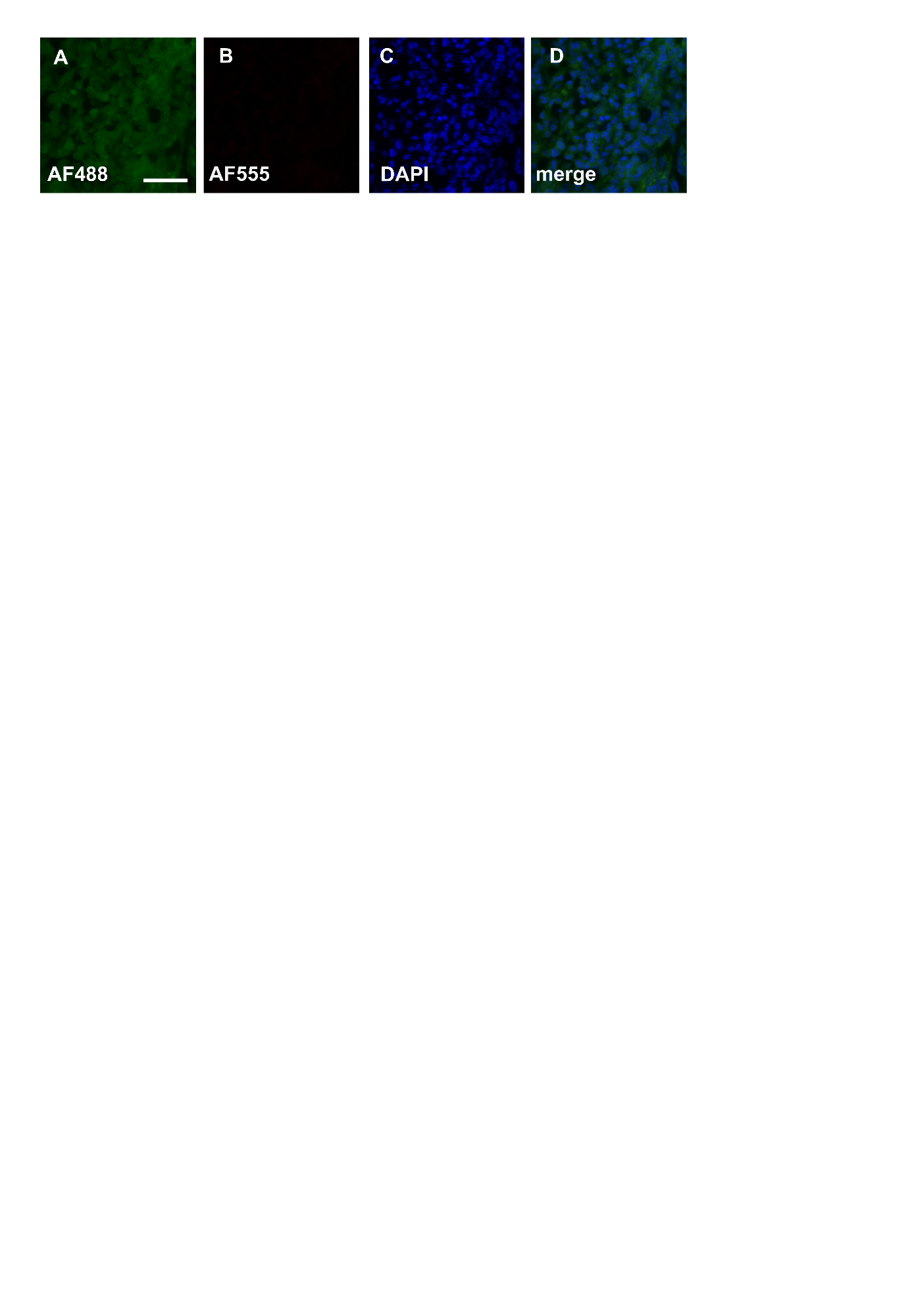
**

Figure S11. Antibody control. Secondary antibodies from goat, α-mouse, Alexa Fluor 488 (A) and α-rabbit, Alexa Fluor 555 (B) were applied without primary antibodies to exclude unspecific binding. Nuclei were stained with DAPI (C). Scale bar: 50 µm.
